# Association between lipid profiles and cigarette smoke among adults in the Persian cohort (Shahedieh) study

**DOI:** 10.1186/s12889-024-18734-0

**Published:** 2024-05-07

**Authors:** Mahdieh Momayyezi, Sara Jambarsang, Hossein Fallahzadeh, Reyhane Sefidkar

**Affiliations:** Center for Healthcare Data Modeling, Department of Biostatistics and Epidemiology, School of Public Health, Shahid Sadoughi University of Medical Sciences, Yazd, Iran

**Keywords:** Smoke, Lipid profiles, Dyslipidemia

## Abstract

**Objectives:**

Exposure to cigarette smoke introduces a large amount of nicotine into the bloodstream through the lungs. So, smoking can be a risk factor for many diseases. The present study was conducted to investigate the effect of active and passive cigarette smoke on the blood lipid profile and dyslipidemia.

**Methods:**

This cross-sectional study was performed on 5052 individuals who participated in the recruitment phase of the Shahedieh cohort study. A logistic regression model was used to investigate the relationship between smoking exposure status and lipid profiles.

**Results:**

The prevalence of abnormal low-density lipoprotein-cholesterol (LDL-C), abnormal HDL-C, abnormal total cholesterol (TC), abnormal triglyceride (TG), and dyslipidemia were 254 (5.00%), 562 (11.10%), 470 (9.30%), 1008 (20.00%), and 1527 (30.20%), respectively. Adjusting for confounders, it was observed that current smokers had higher odds of having abnormal HDL-C [OR (95% CI), 2.90 (2.28–3.69)], abnormal TG [OR (95% CI), 1.71 (1.38–2.13)] and dyslipidemia [OR (95% CI), 1.86 (1.53–2.25)]. Ex-smokers also had greater odds of abnormal HDL-C [OR (95% CI), 1.51 (1.06–2.16)] compared to non-smokers who were not exposed to cigarette smoke.

**Conclusions:**

The findings indicated that current smokers had higher TG and lower HDL. So, necessary measures should be taken to reduce smoking. The findings also showed that the prevalence of abnormal TG and HDL in ex-smokers was lower than in current smokers. Therefore, the existence of incentive policies to quit smoking seems necessary.

## Introduction

Dyslipidemia refers to any disorder in fat metabolism that causes an increase in one or some blood lipid profiles, including triglyceride (TG), total cholesterol (TC), low-density lipoprotein-cholesterol (LDL-C), or a decrease in high-density lipoprotein-cholesterol (HDL-C) [1]. Dyslipidemia is a modifiable risk factor for cardiovascular disease. Its control is effective in reducing cardiovascular diseases. Its prevalence is increasing in developing countries such as Iran, due to the change from traditional lifestyle to modern life and decrease in physical activity [2]. According to the Atlas of STEPwise approach to non-communicable disease (NCD) risk factor surveillance (STEPs) in 2021, 30.58% of the population of Iran and 38.87% of the population of Yazd province had high cholesterol (TC > 200 mg/dl). Also, the prevalence of high LDL (LDL > 160 mg/dl) was 14.96% in Iran and 22.72% in Yazd province [3]. Some studies have shown that smoking is a risk factor for dyslipidemia. It is also an essential risk factors for non-communicable diseases [4].

The long delay between smoking and the onset of smoking-related diseases leads to ignorance of the effects of smoking. Smokers lose an average of one day of their lives per week of smoking. Smoking also leads to the death of one smoker out of three current smokers [5]. Tobacco kills more than 8 million people annually, and 1.2 million of them are non-smokers who were exposed to other people’s cigarette smoke [6]. The WHO report showed that in 2020, 20.3% of the world’s population used tobacco [6]. The results of the STEPS 2021 showed that 9.33% of Iranian people were currently smokers, and 21.2% were non-smokers who were exposed to other people’s cigarette smoke [3].

Some studies have shown that smoking hurts plasma concentrations and blood lipoprotein levels [7–9]. Research on animals indicated that exposure to cigarette smoke disrupts plasma lipid profile and HDL function [10, 11]. Cigarette smoke contains various compounds, including nicotine, which enters the blood circulation through the lungs and harms human health [5]. A meta-analysis study showed that the effect of smoking on triglycerides continued even after quitting smoking, and the researcher recommended that smokers should quit smoking as soon as possible [12]. Since no conclusive association has been established between dyslipidemia and smoking based on the available evidence, we investigated the relationship between exposure to cigarette smoke status and lipid profiles and dyslipidemia in a population-based research conducted in Yazd, Iran.

## Methods

### Study design and population

Data of this study was derived from the recruitment phase of the Shahedieh cohort study which was initiated in 2016 on 10,000 Iranian adults between 35 and 70 years old from three cities of Yazd province (Shahedieh, Zarch, and Ashkezar). Shahedieh study is a part of the PERSIAN (Prospective Epidemiological Research Studies in Iran) multicenter cohort study. More details about the protocol of the PERSIAN cohort study are accessible elsewhere [13].

### Sample size and participants

Of the 10,000 individuals who participated in the Shahedieh study, the information of 676 individuals was incomplete. Furthermore, 4272 individuals who drank alcohol (8.50%) and/or had renal failure (0.80%) and/or had hepatitis (0.62%) and/or had hypertension (21.10%) and/or had diabetes (18%) and/or had fatty liver disorder (10.80%) and/or get medication for lipid disorders (15.10%) were excluded from the analysis [14, 15].

### Definition of Dyslipidemia

In this study, LDL-C ≥ 160 mg/dL and /or TC ≥ 240 mg/dL and/or HDL-C < 40 mg/dL and/or TG > 200 mg/dL was defined as dyslipidemia [14].

### Exposure to cigarette smoke

The subjects were classified into four groups, including smokers, ex-smokers, non-smokers who were exposed to cigarette smoke, and non-smokers who were not exposed to cigarette smoke. Current smokers were defined as people who reported they had smoked at least 100 cigarettes and they were currently smoking regularly or occasionally. The non-smoker category included those who reported they smoked less than 100 cigarettes during their lifetime [14]. Ex-smokers were those who had quit with a history of smoking at least 100 cigarettes. We also define exposure to cigarette smoke as exposure to smoke at home or workplace. Cigarette smoke status was self-reported in the Shahedieh cohort study.

### Covariates

Information on demographic characteristics (e.g., age, sex, marital status, and education level) was obtained through standardized questionnaires. A digital scale (SECA, model 755, Germany) was used to measure weight when the individuals had minimum clothing and were without shoes. The height of participants was also measured with a precision of 0.5 cm with a tape measure that was attached to the wall without any bumps [13]. The physical activity of participants was measured through face-to-face interviews using the International Physical Activity Questionnaire which was translated to Farsi and validated for the Iranian population previously [16]. The results of this Questionnaire were expressed in terms of metabolic equivalents per week (MET-hours per week). In this study, levels of physical activity were determined as low (24- 36.5 MET-hours per week), moderate (36.6–44.9 MET-hours per week), and heavy (≥ 45 MET-hours per week) [14].

### Statistical analysis

In this study, frequency (percentages) were used to describe variables. The Chi-square test and the linear trend test were used to compare the baseline characteristics of smokers, ex-smokers, non-smokers who were exposed to cigarette smoke, and non-smokers who were not exposed to cigarette smoke. Abnormal blood lipids were also compared among individuals with different levels of exposure to cigarette smoke. In this study, the crude and adjusted odds ratios (ORs) and 95% confidence intervals (CIs) were also estimated to examine the relationship between smoking exposure status and the odds of abnormal blood lipids and dyslipidemia using logistic regression. To estimate adjusted ORs, the baseline characteristics (age, marital status, education, MET-hours per week, and BMI) with more than o.3 p-value in univariate analysis were included in the final model. Analysis was performed through SPSS (version 26) software.

## Results

Among 5052 individuals who entered the recruitment phase of the Shahedieh cohort study, 2599 (51.40%) were female, 3734 (73.90%) were in the age range of 35–50 years, 4907 (97.10%) were married, 1494 (26.90%) were in secondary level, 2770 (54.80%) had moderate physical activity, 2161 (42.80%) were overweight. Based on the results of Table 1, The relation between gender, age, marital status, education, physical activity, body mass index, and smoking exposure status is significant (*p* < 0.001).

**Table 1:** Demographic characteristics of current smokers, ex-smokers, non-smokers who were exposed to cigarette smoke, and non-smokers who were not exposed to cigarette smoke

Variables	Total (*N* = 5052)	Non-smoker and non-exposed (*N* = 2697)	Non-smoker but exposed (*N* = 1439)	Ex-smoker (*N* = 318)	Current Smoker (*N* = 598)	*P*
**Sex**						
Male	2453 (48.60%)	1065 (39.50%)	478 (33.20%)	318 (100%)	592 (99%)	*P* < 0.001
Female	2599 (51.40%)	1632 (60.50%)	961 (66.80%)	0 (0%)	6 (1.00%)	
**Age (years)**						
35–50	3734 (73.90%)	2056 (76.20%)	1111 (77.20%)	164 (51.60%)	403 (67.40%)	
51–65	1193 (23.60%)	566 (21.00%)	306 (21.30%)	139 (43.70%)	182 (30.40%)	*P* < 0.001
> 65	125 (2.50%)	75 (2.80%)	22 (1.50%)	15 (4.70%)	13 (2.20%)	
**Marital status**						
Single	22 (0.40%)	13 (0.50%)	7 (0.50%)	2 (0.60%)	0 (0%)	
Married	4907 (97.10%)	2621 (97.20%)	1374 (95.50%)	315 (99.10%)	597 (99.80%)	*P* < 0.001
Widow/ Divorced	123 (2.50%)	63 (2.30%)	58 (4%)	1 (0.30%)	1 (0.20%)	
**Educational Status**						
Illiterate	502 (9.90%)	246 (9.12%)	157 (10.90%)	40 (12.57%)	59 (9.87%)	
Primary	1494 (26.90%)	753 (27.92%)	485 (33.70%)	81 (25.50%)	175 (29.26%)	
Intermediate	915 (18.10%)	451 (16.72%)	256 (17.80%)	75 (23.58%)	133 (22.24%)	*P* < 0.001
Secondary	1188 (23.50%)	672 (24.92%)	298 (20.70%)	77 (24.20%)	141 (23.58%)	
Higher	953(18.90%)	575 (21.32%)	243 (16.90%)	45 (14.15%)	90 (15.05%)	
**Physical activity (METs-hours per week)**						
24-36.5	1001 (19.80%)	496 (18.40%)	293 (20.40%)	74 (23.30%)	138 (23.10%)	
36.6–44.9	2770 (54.80%)	1561 (57.90%)	845 (58.70%)	124 (39.00%)	240 (40.10%)	*P* < 0.001
≥ 45	1281 (25.40%)	640 (23.70%)	301 (20.90%)	120 (37.70%)	220 (36.80%)	
**BMI**						
≤ 18.50	96 (1.90%)	49 (1.80%)	14 (1.00%)	6 (1.90%)	27 (4.50%)	
18.60–24.90	1363 (27%)	689 (25.50%)	339 (23.55%)	93 (29.25%)	242 (40.50%)	
25–29	2161 (42.80%)	1183 (43.90%)	593 (41.20%)	158 (49.70%)	227 (38.00%)	*P* < 0.001
30-34.90	1098 (21.70%)	598 (22.20%)	367 (25.50%)	51 (16.05%)	82 (13.70%)	
≥ 35	334 (6.60%)	178 (6.60%)	126 (8.75%)	10 (3.10%)	20 (3.30%)	

The prevalence of abnormal LDL-C, abnormal HDL-C, abnormal TC, abnormal TG, and dyslipidemia were 254 (5.00%), 562 (11.10%), 470 (9.30%), 1008 (20.00%), and 1527 (30.20%), respectively. The LDL/HDL and TC/HDL mean were 2.07 (0.69) and 3.70 (1.02). It was also observed that the relationship between abnormal HDL-C (*p* < 0.001), abnormal TG (*p* < 0.001), dyslipidemia (*p* < 0.001) and smoking status was significant. The prevalence of abnormal HDL, abnormal TG, and dyslipidemia were higher in smokers (22.10%, 26.10%, and 40.00%) and ex-smokers (13.50%, 22.30%, and 31.40%) compared to non-smokers who were exposed to cigarette smoke (9.20%, 18.30% and 28.60%) and non-smokers who were not exposed to cigarette smoke (11.10%, 20.00% and 30.20%). The LDL/HDL (*p* < 0.001) and TC/HDL (*p* < 0.001) means were also significantly different in terms of smoking status. Furthermore, the LDL/HDL and TC/HDL mean of smokers and ex-smokers was higher than both non-smoker groups (Table 2).

**Table 2:** Lipid profile of smokers, ex-smokers, non-smokers who were exposed to cigarette smoke, and non-smokers who were not exposed to cigarette smoke

	Total (*N* = 5052)	Non-smoker and non-exposed (*N* = 2697)	Non-smoker but exposed (*N* = 1439)	Ex-smoker (*N* = 318)	Current smoker (*N* = 598)	*P*
**Abnormal LDL**						
No	4798 (95.00%)	2561 (95.00%)	1365 (94.90%)	303 (95.30%)	569 (95.20%)	0.98
Yes	254 (5.00%)	136 (5.00%)	74 (5.10%)	15 (4.70%)	29 (4.80%)	
**Abnormal HDL**						
No	4490 (88.90%)	2443 (90.60%)	1306 (90.80%)	275 (86.50%)	466 (77.90%)	*P* < 0.001
Yes	562 (11.10%)	254 (9.40%)	133 (9.20%)	43 (13.50%)	132 (22.10%)	
**Abnormal TC**						
No	4582 (90.70%)	2443 (90.60%)	1298 (90.20%)	294 (92.50%)	547 (91.50%)	0.56
Yes	470 (9.30%)	254 (9.40%)	141 (9.80%)	24 (7.50%)	51 (8.50%)	
**Abnormal TG**						
No	4044 (80.00%)	2179 (80.80%)	1176 (81.70%)	247 (77.70%)	442 (73.90%)	*P* < 0.001
Yes	1008 (20.00%)	518 (19.20%)	263 (18.30%)	71 (22.30%)	156 (26.10%)	
**Dyslipidemia**						
No	3525 (69.80%)	1921 (71.20%)	1027 (71.40%)	218 (68.60%)	359 (60.00%)	*P* < 0.001
Yes	1527 (30.20%)	776 (28.80%)	412 (28.60%)	100 (31.40%)	239 (40.00%)	
LDL/HDL	2.07 (0.69)	2.03 (0.68)	2.02 (0.68)	2.23 (0.73)	2.25 (0.73)	*P* < 0.001
TC/HDL	3.70 (1.02)	3.65 (1.00)	3.61 (0.96)	3.96 (1.12)	4.04 (1.10)	*P* < 0.001

Adjusting for age, marital status, education, MET-hours per week, and BMI, it was observed that current smokers had higher odds of having abnormal HDL-C [OR (95% CI), 2.90 (2.28–3.69)], abnormal TG [OR (95% CI), 1.71 (1.38–2.13)] and dyslipidemia [OR (95% CI), 1.86 (1.53–2.25)]. Ex-smokers also had greater odds of abnormal HDL-C [OR (95% CI), 1.51 (1.06–2.16)] compared to non-smokers who were not exposed to cigarette smoke (Table 3).

**Table 3:** ORs (95% CIs) in smoking status for abnormal lipid profiles and dyslipidemia status

Abnormal lipid profiles and dyslipidemia	Cigarette smoke status	OR (95% CI)*	OR (95% CI)**
**Abnormal LDL**	Current smoker	0.86(0.64,1.15)	1.07(0.70,1.62)
	Ex-smoker	0.83(0.57,1.20)	0.87(0.49,1.51)
	Non-smoker but exposed	1(0.80,1.24)	0.98(0.73,1.32)
**Abnormal HDL**	Current smoker	2.78(2.37,3.27)	**2.90 (2.28–3.69)**
	Ex-smoker	1.74(1.40,2.16)	**1.51 (1.06–2.16)**
	Non-smoker but exposed	0.98(0.83,1.15)	0.99 (0.79–1.24)
**Abnormal TC**	Current smoker	0.87(0.71,1.08)	0.98(0.71,1.36)
	Ex-smoker	0.73(0.55,0.97)	0.71(0.45,1.11)
	Non-smoker but exposed	1.16(0.99,1.35)	1(0.80,1.24)
**Abnormal TG**	Current smoker	1.40(1.23,1.61)	**1.71 (1.38–2.13)**
	Ex-smoker	1.10(0.92,1.31)	1.18 (0.88–1.57)
	Non-smoker but exposed	1.02(0.91,1.15)	0.93 (0.781.10)
**Dyslipidemia**	Current smoker	1.56(1.37,1.76)	**1.86 (1.53–2.25)**
	Ex-smoker	1.15(0.98,1.35)	1.08 (0.83–1.40)
	Non-smoker but exposed	1.03(0.93,1.14)	0.97 (0.84–1.12)

^*^Crude model of the association between cigarette smoke status and abnormal lipid profiles; reference group is the non-smoker and non-exposed group

^**^ Adjusted for age, marital status, education, MET-hours per week, and BMI for abnormal HDL, abnormal TG, and dyslipidemia. Adjusted for age, marital status, education, and BMI for abnormal LDL, and abnormal TC; reference group is the non-smoker and non-exposed group

## Discussion

The prevalence of dyslipidemia in the present study was 30%, which was lower than the studies with the similar dyslipidemia definition [14, 17]. This difference could be attributed to their good physical activity, more than 80% of individuals had moderate level and above, which may be related to their occupation. Shahedieh study was conducted in an urban area which most individuals were involved in animal farming and agriculture. As a result, the lower prevalence of dyslipidemia observed in this study is probably related to the participants’ occupations and associated lifestyle factors.

Based on the results, a notable relation was found between dyslipidemia and smoking habits. The prevalence of dyslipidemia was higher among the current smokers and the ex-smokers compared to non-smokers who were exposed and non-smokers who were not exposed. Furthermore, the odds of dyslipidemia in the current smokers was 86% more than those who never smoked and also were not exposed to smoke, which is consistent with the Ma et al. study that demonstrated cigarette smoke disrupted lipid metabolism [18]. Another study also reported that smoking increase the odds of dyslipidemia by 2.53% [19]. Furthermore, other human and animal studies have shown that exposure to cigarette smoke significantly increases TG, TC, and LDL and reduces HDL [20–22]. The mentioned studies demonstrated the negative impact of cigarette smoke on lipid metabolism and overall cardiovascular health. It was observed that smoking disrupts the balance of lipids in the body, leading to increased levels of TG, TC, and LDL and causing HDL to be decreased. This imbalance or dyslipidemia increases the risk of developing cardiovascular diseases. It is crucial to understand these consequences to encourage smokers to quit and promote a healthier lifestyle for the well-being of individuals and society.

There are many discussions about which components of lipid profiles are mainly changed by smoking. Current study highlights a significant association between abnormal TG and smoking habits. The prevalence of abnormal TG is notably higher in current smokers compared to non-smokers. The logistic regression model indicated that the odds of having abnormal TG is 71% greater in current smokers than in non-smokers who were non-exposed. Al-Jaf et al. reported that the prevalence of abnormal TG and LDL in smokers is significantly higher than non-smokers [5]. Jain et al. showed that the prevalence of abnormal TG and HDL in smokers was significantly higher than non-smokers [23]. These findings underscore the possible impacts of smoking on various components of lipid profiles. Jain et al. also showed that the relationship between abnormal TG and HDL with smoking was significant among the lipid profiles. The adjusted odds of abnormal TG in current smokers was 1.3% of non-smokers in their study [23]. A review of other studies showed that in most studies, cigarette smoke had a significant effect on TG [5, 8, 22, 24, 25]. In the study of Kim et al., among the lipid profiles, only TG was significantly associated with smoking, and the prevalence of abnormal TG in current smokers and ex-smokers was more than in non-smokers [26].

In the present study, the prevalence of abnormal TG in the current smokers was more than ex-smokers. Current smokers are more likely to have abnormal TG compared to ex-smokers due to the direct effects of smoking on lipid metabolism, inflammation, and oxidative stress. On the other hand, ex-smokers who have quit smoking for a while may experience balance in their lipid profile as their body recovers from the effects of smoking. However, the extent of these improvements may vary depending on factors like the duration since quitting and the overall health status of the individual. Quitting smoking can significantly reduce the risk of abnormal TG and improve overall health. Attard et al. results supports the notion that quit smoking can help reduce TG [27]. A meta-analysis study found that the impact of smoking on TG persists even after quitting [12]. Smokers’ diets may contain more fat and less fiber and grains than non-smokers, contributing to elevated levels of detrimental blood fats [28] which can be a confounder of the current results.

This study highlights a significant association between abnormal HDL and smoking habits. The prevalence of abnormal HDL in current smokers and ex-smokers were higher than others. The results of this study showed that the odds of abnormal HDL was 90% in current smokers and 51% in ex-smokers higher than non-smokers and non-exposed group. Animal studies have indeed demonstrated that exposure to cigarette smoke can lead to a decrease in HDL (high-density lipoprotein), often referred to as “good cholesterol.” [11, 21]. In another laboratory based study, it was observed that exposure to cigarette smoke reduced HDL of mice by 22% [21]. The results of human studies also indicate the adjusted odds of abnormal HDL in current smokers was 1.6% of non-smokers [23]. A meta-analysis has shown that both active and passive smoking can negatively impact HDL [4]. This is due to several factors, including higher homocysteine levels in smokers, which hurts HDL [29]. Additionally, smoking may reduce estrogen levels, leading to a decrease in HDL [30]. These findings suggest that smoking may contribute to a decline in HDL, which can have adverse effects on overall cardiovascular health. It is worth to mention that the association between LDL and TC with smoking exposure status was not significant in this study.

Although this study was carried out on a large, representative sample of Iranian adults, it had some limitations that merit to be mentioned. Since the design of this study was cross-sectional, we merely studied the association between smoking habits and lipid profile levels and we could not derive causal inferences on this matter. The second limitation is related to the potential bias for smoking exposure status as it is self-reported. The absence of recording details about the amount and duration of cigarette usage among the participants in the Shahedieh Cohort study was another limitation. This lack of information makes it difficult to accurately compare the intensity of exposure to cigarette smoke with an individual’s lipid blood profile.

## Conclusion

As shown in the present research, smoking exposure has adverse effects on TG and HDL. The results indicated that current smokers had higher TG and lower HDL. So, necessary health recommendations is needed to reduce smoking. The findings also showed that the prevalence of abnormal TG and HDL in ex-smokers was lower than in current smokers. Therefore, even quitting smoking can have an acceptable effect in people who have been smoking for a while. Also, considering that the prevalence of abnormal TG and HDL in current smokers was higher than in people who were exposed to cigarette smoke. Short educational videos can be an effective tool in raising awareness and encouraging people to quit smoking, ultimately balancing their lipid blood profiles and overall health.

## Data Availability

No datasets were generated or analysed during the current study.
